# Increased mortality attributed to Chagas disease: a systematic review and meta-analysis

**DOI:** 10.1186/s13071-016-1315-x

**Published:** 2016-01-27

**Authors:** Zulma M. Cucunubá, Omolade Okuwoga, María-Gloria Basáñez, Pierre Nouvellet

**Affiliations:** London Centre for Neglected Tropical Disease Research (LCNTDR), Department of Infectious Disease Epidemiology, School of Public Health, Faculty of Medicine (St Mary’s campus), Imperial College London, Norfolk Place, London, W2 1PG United Kingdom; Grupo de Parasitología – RED CHAGAS, Instituto Nacional de Salud, Bogotá, Colombia; Medical Research Council Centre for Outbreak Analysis and Modelling, Department of Infectious Disease Epidemiology, School of Public Health, Faculty of Medicine (St Mary’s campus), Imperial College London, London, UK

**Keywords:** Chagas disease, *Trypanosoma cruzi*, Cardiomyopathy, Survival, Excess mortality, Mortality rate, Relative risk, Attributable risk, Meta-analysis, Heterogeneity

## Abstract

**Background:**

The clinical outcomes associated with Chagas disease remain poorly understood. In addition to the burden of morbidity, the burden of mortality due to *Trypanosoma cruzi* infection can be substantial, yet its quantification has eluded rigorous scrutiny. This is partly due to considerable heterogeneity between studies, which can influence the resulting estimates. There is a pressing need for accurate estimates of mortality due to Chagas disease that can be used to improve mathematical modelling, burden of disease evaluations, and cost-effectiveness studies.

**Methods:**

A systematic literature review was conducted to select observational studies comparing mortality in populations with and without a diagnosis of Chagas disease using the PubMed, MEDLINE, EMBASE, Web of Science and LILACS databases, without restrictions on language or date of publication. The primary outcome of interest was mortality (as all-cause mortality, sudden cardiac death, heart transplant or cardiovascular deaths). Data were analysed using a random-effects model to obtain the relative risk (RR) of mortality, the attributable risk percent (ARP), and the annual mortality rates (AMR). The statistic I^2^ (proportion of variance in the meta-analysis due to study heterogeneity) was calculated. Sensitivity analyses and publication bias test were also conducted.

**Results:**

Twenty five studies were selected for quantitative analysis, providing data on 10,638 patients, 53,346 patient-years of follow-up, and 2739 events. Pooled estimates revealed that Chagas disease patients have significantly higher AMR compared with non-Chagas disease patients (0.18 versus 0.10; RR = 1.74, 95 % CI 1.49–2.03). Substantial heterogeneity was found among studies (I^2^ = 67.3 %). The ARP above background mortality was 42.5 %. Through a sub-analysis patients were classified by clinical group (severe, moderate, asymptomatic). While RR did not differ significantly between clinical groups, important differences in AMR were found: AMR = 0.43 in Chagas vs. 0.29 in non-Chagas patients (RR = 1.40, 95 % CI 1.21–1.62) in the severe group; AMR = 0.16 (Chagas) vs. 0.08 (non-Chagas) (RR = 2.10, 95 % CI 1.52-2.91) in the moderate group, and AMR = 0.02 vs. 0.01 (RR = 1.42, 95 % CI 1.14–1.77) in the asymptomatic group. Meta-regression showed no evidence of study-level covariates on the effect size. Publication bias was not statistically significant (Egger's test *p*=0.08).

**Conclusions:**

The results indicate a statistically significant excess of mortality due to Chagas disease that is shared among both symptomatic and asymptomatic populations.

**Electronic supplementary material:**

The online version of this article (doi:10.1186/s13071-016-1315-x) contains supplementary material, which is available to authorized users.

## Background

Chagas disease is a neglected tropical disease (NTD) of global health concern with around 13 % of the population residing in Latin America considered at risk of *Trypanosoma cruzi* (Kinetoplastida: Trypanosomatidae) infection. The infection is endemic in 21 countries [1]. Currently, it is estimated that Chagas disease affects between 6 and 8 million individuals, with an attributed number of deaths of approximately 12,000 per year worldwide [2].

Although Chagas disease was first described more than a century ago, the course of the disease and its clinical outcomes are still not well understood [3]. The clinical course of Chagas disease is usually divided into acute and chronic phases. In most cases, the initial infection is asymptomatic. However, a few cases will present acute symptoms and in some instances death may occur [4, 5]. Infected individuals surviving the acute phase―which is the most common occurrence―enter the indeterminate stage, characterised by a long asymptomatic period before the onset of clinical signs and symptoms. The latter can last 10–30 years or until the end of an individual’s life [6]. Based on early cohort studies, an estimated 20-30 % of infected individuals would eventually develop heart disease, with an associated increased mortality [5, 7, 8]. A recent cohort study on infected blood donors in Brazil showed an annual rate of progression to cardiomyopathy of 1.85 % per year [9] and other studies have found that Chagas disease is an independent risk factor for stroke [10, 11]. Although Chagas disease is one of the NTDs globally with a large proportional contribution of years of life lost (YLL) to its total disability-adjusted life years (DALYs) [12], there is a paucity of research measuring rigorously disease progression rates according to the different stages described above and quantifying excess mortality due to Chagas disease compared with mortality rates in non-chagasic individuals.

Processes of extensive inflammation and fibrosis appear to be involved in the physiopathology of the chagasic cardiomyopathy [7]. The clinical manifestations of Chagas disease may be characterised by the grade of myocardial damage [13]. However, although some similarities in clinical presentation occur, there is evidence that Chagas cardiomyopathy has specific characteristics that could influence mortality when compared with other aetiologies or with idiopathic dilated cardiomyopathy [14].

A better understanding of the magnitude of morbidity and mortality associated with Chagas disease is imperative for appropriately measuring burden of disease and evaluating the cost effectiveness of strategies to prevent and control *T. cruzi* infection and its clinical sequelae. This is because mathematical modelling of infection and disease, ensuing burden of disease quantifications, and accompanying cost-effectiveness studies critically rely on estimates of Chagas disease-related morbidity and mortality rates [2, 15, 16]. However, inspection of the literature reveals a great deal of variation in the reported mortality rates attributed to Chagas disease, raising difficulties of interpretation and hampering their use in model parameterisation.

In 2007, Rassi et al. [17] reviewed predictors of mortality in chronic Chagas disease, reporting annual mortality rates from a selection of papers, which ranged from 0.2 to 19.2 % per year without a control group comparison. More recently, and specifically for sudden death, De Souza et al. [18] found, in a retrospective cohort study, that mortality rates varied according to the clinical severity of the chronic phase, being estimated as 1.5 %, 25 %, and 51 %, for mild, moderate and severe cases, respectively (see also Rassi et al. [19]). The inconsistency of reported rates is likely to be the result of heterogeneity in the clinical presentation of the populations studied. Additionally, Linetzky et al. [20].conducted a systematic review comparing cardiovascular outcomes between Chagas and non-Chagas patients, and reported higher mortality risk in chagasic patients but without quantifying the effect.

In view of the above, the objectives of the present study are: 1) to conduct a systematic review of the literature in order to identify those studies in which mortality was measured for both chagasic and non-chagasic (control) patients, 2) to perform a meta-analysis of such studies in order to derive estimates of risk ratios, attributable risk, and mortality rates associated with Chagas disease, and 3) to provide overall and stage-specific estimates of mortality for further mathematical modelling of the relationship between infection and morbi-mortality, burden of disease estimation and economic evaluations.

## Methods

The PRISMA (Preferred Reporting Items for Systematic Reviews and Meta-Analyses) guidelines and checklist was used to ensure inclusion of relevant information in the analysis [21] (Additional file 1).

### Search strategies

Searches were conducted in PubMed (online version of Index Medicus, produced by the USA National Library of Medicine, NLM); MEDLINE (a subset of PubMed (~98 %) made available by NLM); EMBASE (Excerpta Medica dataBASE), Web of Science (Core Collection) and LILACS (Latin American and Caribbean Health database), without time filters until the 31^st^ of September 2015. The search algorithm combined four search terms to represent the grouping of the concepts most relevant to the question under scrutiny: 1) Chagas disease, 2) mortality, 3) progression, and 4) survival analysis. This search algorithm was applied to each database to maintain consistency in the results generated. The full search terms for individual databases are available in Additional file 2: Table S1. All the titles and abstracts were assessed by two independent investigators (ZMC and OO), eliminating studies that did not meet the inclusion criteria: i. cohort studies, ii) comparing Chagas and non-Chagas patients, iii) with follow-up for more than one year. Disagreements were resolved by consensus, and in the case of persistent disagreement, the full text of the article was examined. References cited in the selected papers were inspected and if appropriate included as secondary searches.

### Data extraction

Each paper that was selected for analysis of the full text was reviewed carefully and the relevant information was extracted. In some instances information was extracted from available data tables or figures, where values were not explicitly mentioned in the text. A data extraction table was designed to obtain information from each eligible study. The following items were included: first author; year of publication; year of study; location of the study; study design; sample size; proportion of men in the study population; age group; mean/median age of study participants; number of deaths; years of follow up; number of persons-year of follow up; loss to follow up (drop-out rate); clinical classification (severe, moderate, asymptomatic); reported effect size and corresponding adjustments.

In order to obtain results accounting for severity of symptoms, the data were extracted and classified according to the clinical severity reported in each study, as follows:

- Severe stage: this stage included patients with cardiac complications, attending health facilities and usually classified according to the New York Heart Association Functional Classification (NYHA) III and IV. Also one study [22] which included only a population under resynchronization therapy was considered in this stage.
- Moderate stage: this included populations mostly classified according to NYHA I and II criteria.
- Asymptomatic/general population: this category included both asymptomatic populations―mainly from population studies―and also individuals with minimal electrocardiogram (ECG) damage or without report of deleterious ejection fraction.
- All stages: this category included studies in which several clinical stages were used in comparison to clinically similar but uninfected controls.

### Quality assessment

The Newcastle-Ottawa Scale (NOS) was used to assess the risk of bias of the studies included in this review in a standardised manner, as this metric is easy to interpret and is recommended for quality assessment by the Cochrane Collaboration [23]. The NOS scale assesses each study on three components, namely, the selection of the study population, a valuation of comparability of the study groups, and an assessment of the outcome of interest. Each study is scored for each component by the award of “stars”. The checklist, amendments made to the original scale and details on the assessment for each study are presented in Additional file 2: Table S2. The critical appraisal of the studies was conducted following the data extraction process. Three levels of quality were considered: low, moderate and high. Due to the small number of studies identified, studies were not excluded based on quality assessment. Nevertheless, a separate analysis was done only using papers deemed as of “high quality”.

### Statistical analysis

Studies were required to report hazard ratios (HRs), relative risk ratios (RRs), odds ratios (ORs) and their 95 % confidence intervals (CIs) or to provide adequate data to allow the 95 % CI to be calculated. Because not all studies reported deaths in a uniform manner, the analysis is based on all-cause mortality, cardiac death, heart transplant or death due to stroke. For quantitative analysis, studies were included if enough information was provided to estimate crude RRs.

Selected studies differed substantially in terms of sample size, study location and clinical characteristics; therefore, heterogeneity in mortality rates was potentially important. Thus, a random-effects model was used to test differences in rates of mortality between chagasic and non-chagasic populations. For the random-effects model, tau-squared (*τ*^2^) was presented as a measure of the between-study variance. For comparison, results using a fixed-effects model are also presented (Additional file 2: Figure S1).

Heterogeneity among studies was measured using Cochran's Q test and I^2^ statistic. Cochran's Q is computed by summing the squared deviations of each study’s estimate from the overall estimate, weighting each study’s contribution. The p-values for this test are obtained by comparing the Q statistic to a chi square distribution with *k*–1 degrees of freedom (df) (where *k* is the number of studies). The I^2^ statistic measures the degree of inconsistency in the studies’ results. Formally, I^2^ = 100 % × (Q–df)/Q, measuring the percentage of variation across studies that is due to heterogeneity rather than to chance [24].

To explore further the source of potential heterogeneity in mortality between studies, we used meta-regression techniques to formally identify potential covariates of the estimated effect on mortality rates [25, 26]. Covariates tested included clinical characteristics (as defined above), starting year of the study, sex (as proportion of males), and location of study (country).

We explored publication bias by drawing funnel plots, enabling quantification of bias using Egger’s regression asymmetry test [27]. Interpretation of funnel plots is facilitated by inclusion of diagonal lines representing the 95 % confidence limits around the summary effect. In the absence of heterogeneity, 95 % of the studies should lie within the funnel defined by these lines (because these are not strictly speaking 95 % limits, they are referred to as “pseudo 95 % confidence limits”) [28]. A trim-and-fill technique (aimed both at identifying and correcting funnel plot asymmetry) was then used to re-estimate excess mortality correcting for publication bias (i.e., by incorporating the hypothetically missing studies) [29].

Finally, sensitivity analyses were also performed by 1) sequentially removing one study at a time and re-evaluating the model to explore the impact of potential outliers on estimates of excess mortality, and 2) restricting the analysis to ‘high quality’ papers.

The crude mortality rates were calculated for each clinical group and RR values were used for meta-analysis. Annual mortality rates (AMR) are reported (unless otherwise stated) per person per year. The Attributable Risk Percent (ARP) was used to estimate excess mortality above background mortality rate, as (RR − 1)/RR expressed in percent.

All analyses were performed using Stata 13.1 (StataCorp, College Station, TX).

## Results

### Description of included studies and data obtained

**Fig. 1:**
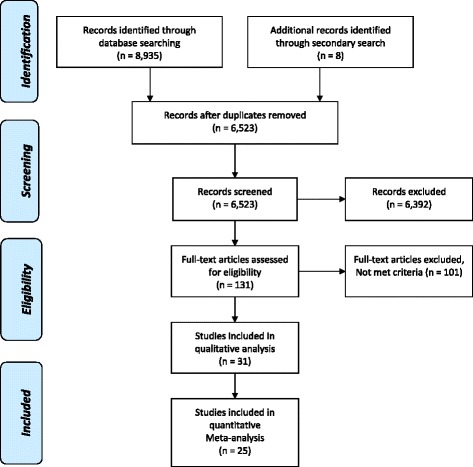
Flow diagram describing the selection of studies included in the meta-analysis

The systematic literature search (Fig. 1), yielded 6523 distinct publications, which were identified and reviewed. After exclusions, 31 entries were selected for inclusion for qualitative analysis. Twenty seven out of the 31 studies were conducted in Brazil; the remaining four were conducted in Bolivia, Chile, United States of America, and Venezuela, each country contributing one study. A total of six studies were excluded from quantitative analysis due to these reports either not providing enough information in the paper [30–32], the outcome was not clearly measured as part of the study [33, 34] or due to the absence of deaths during the follow-up period [35]. A total of 25 were selected for quantitative meta-analysis, of which 12 (48 %) were classified as of high quality, 11 (44 %) as of moderate quality, and 2 (8 %) as of low quality. Detailed results on quality assessment of the 25 studies are presented in Additional file 2: Table S2. These studies yielded data on 10,638 patients, 53,346 patient-years of follow-up, and 2739 events.

Only 17 out of the selected 25 studies provided actual metrics for the relationship between Chagas disease and death, all of them reporting a positive effect (RR, HR or OR greater than 1). Only 9 of these studies adjusted for covariates such as age, sex, other risk factors, schooling, etc. The complete list of studies included in the quantitative analysis is provided in Table 1.

**Table 1:** Characteristics of the 25 studies included in the meta-analysis of Chagas disease-associated mortality

First author, year [Ref]	Country	Outcome	Study period	Diagnostic test	Population type	Control group	Mode of death	Disease classification	Sample size	Outcome	Person-yrs of follow up	Crude RR	Reported effect estimate	
									exp/ non-exp	exp/non-exp	exp/non-exp		Estimate (95% CI)	Adjusted by variables
Coura, 1985 [51]	Brazil	Death	1974-1984	Serology	General population endemic area (included undetermined and cardio-myopathy)	Same population but uninfected	Cardio-myopathy	All stages	235/216	54/23	2350/2160	2.16	2 times higher (NR)	NR
Pereira, 1985 [52]	Brazil	Death	1976-1980	IFAT & CF	General population - Municipality La Lapa	General population - same municipality	SD (64.7%)	Asymptomatic/GenP	192/188	22/6	1152/1128	3.59	NR(NR)	NR
Maguire, 1987 [53]	Brazil	Death	1974-1980	IFAT & CF & ELISA	Asymptomatic from rural Brazilian community. Normal ECG	Asymptomatic from rural Brazilian community. Normal ECG	NR	Asymptomatic/GenP	40/116	3/3	949/771	0.81	RR=1.8 (0.8–4.2)	NR
Mota, 1990 [54]	Brazil	Death	1974-1983	IFAT & CF & ELISA	Rural population	Rural population, slightly healthier	NR	Asymptomatic/GenP	488/509	34/28	3842/3663	1.16	RR=1.1 (NR)	Age adjusted
Bestetti & Muccillo, 1997 [8]	Brazil	Cardiac death	1990-1993	NR	Left ventricular dilatation in ECG. Cardiomegaly in the chest X-ray. With or without symptoms	Similar, uninfected. HTA 34%	SD (38%)	Moderate stage	75/50	21/3	225/150	4.67	OR=6.1 (1.7–21.7)	Not adjusted
Pimenta & Valente, 1999 [55]	Brazil	Cardiac death	1977 1996	CF & IFAT & HAI	Asymptomatic individuals with bundle branch block (RBBB = 98.2%)	Sclerosis of the conducting system of the heart (Lev-Lenègre’s disease) (RBBB: 48.3%)	SD (50%)	Asymptomatic/GenP	55/29	17/3	554/229	2.34	NR (NR)	NR
Freitas, 2005 [56]	Brazil	Death	1991-2000	Serology	NYHA III or IV	IDC	NR	Severe stage	242/454	110/156	516/968	1.32	HR=1.63 (1.10–1.43)	NR
Oliveira, 2005 [57]	Brazil	Death	1993-1995	NR	Systolic ventricular dysfunction (LVEF <55%) at the day of hospitalisation. Chagas in 44% of patients	Any aetiology identified	NR	Severe stage	56/70	50/51	71/89	1.23	RR=2.66 (1.10–6.46)	Not adjusted
De Campos Lopes, 2006 [49]	Brazil	Cardiac death	1998-2000	NR	Severe HF; hospitalized subsequent-ly. HT was considered a censored event	Hospitalized from the same clinic	NR	Severe stage	102/392	72/169	204/784	1.64	NR (NR)	Age and controlled for relevant covariates: health system, myocardial infarction, HTA
Heringer-Walther, 2006 [58]	Brazil	Cardiac death or HT	2001-2006	2 positive serologies	Dilated cardio-myopathy. All stages	Idiopathic dilated cardio-myopathy. Other structural cardiac diseases and comor-bidities were excluded in both groups	NR	All stages	274/504	8/10	716/1108	1.24	OR=3.34 (1.90–5.89)	NR
Braga, 2008 [59]	Brazil	Death	2003-2004	NR	HF and moderate to severe left ventricle systolic dysfunction	Other aetiologies not specified	NR	Moderate stage	89/102	16/10	89/102	1.83	OR=1.67 (0.67–4.41)	Education level
Silva, 2008 [60]	Brazil	Death	NR	NR	Admitted for decom-pensated HF. NYHA III-IIV	Other aetiologies	NR	Severe stage	122/232	84/111	122/232	1.44	NR (NR)	NR
Lima-Costa 2010a [61]	Brazil	Death	1997-2007	HAI & 2 ELISA tests	Bambui, general >60 years age	General population > 60 years age	NR	Asymptomatic/GenP	524/874	257/310	4569/7621	1.38	NR, p<0.01	Age, sex, and a number of risk factors
Lima-Costa, 2010b [50]	Brazil	Stroke	1997-2007	HAI & 2 ELISA tests	Bambui, general population >60 years age (RBBB in 23.5%, in schooled = 20%)	General population > 60 years of age and significantly healthier than chagasic patients (RBBB in 3.3%) and schooled (50%)	Stroke	Asymptomatic/GenP	563/915	20/25	3479/6261	1.40	HR=1.56 (1.32–1.85)	Age, sex, schooling, other risk factors and C-reactive protein level
Nunes, 2010 [62]	Brazil	Cardiac death or HT	1999-2008	NR	HF, dilated cardio-myopathy (diameter/ body surface area ≥31 mm) and LVEF <55%. NYHA III/IV in 25%	Same criteria but Idiopathic dilated cardio-myopathy (NYHA III/IV 27%)	Progressive HF (48%). SD (42%)	Moderate stage	224/63	91/22	737/207	1.16	HR=2.35 (1.25–4.44)	NR
Issa, 2010 [40]	Brazil	Death or HT	1999-2000	IFAT & HAI & ELISA	Clinical trial. Irreversible chronic HF of at least 6-month duration	Other aetiologies, not specified	NR	All stages	68/388	49/180	250/1428	1.55	NR (NR)	Cox proportional hazards regression model
Cardoso, 2010 [63]	Brazil	Death	2006-2007	ELISA & IFAT	NYHA IV, admitted; poor perfusion and congestion. (LVEF) < 45.0%	HTA, Idiopathic, vasculopathic; alcohol cardiomyopathy	Progressive HF	Severe stage	33/67	22/24	68/139	1.87	HR=2.48 (1.28–4.78)	Multi-variate analysis
Conceição-Souza, 2010 [64]	Brazil	Death	2008-2010	NR	(LVEF) < 45.0%. Onset of symptoms>1 month	Same criteria and excluded co-morbidities	NR	Moderate stage	100/62	6/2	100/62	1.86	NR (NR)	NR
Cruz, 2010 [65]	Brazil	Cardiac death	NR	NR	HF; clinics, patients under maximal tolerated medical treatment	IDC (33%), HTA (13%), ischemic (12%)	SD and progressive HF	Moderate stage	21/55	7/11	23/61	1.69	RR=2.75 (1.35–5.63)	NR
Barbosa, 2011 [66]	Brazil	Death or HT	2000-2008	Serology	LVEF <55% in ECG or <50% on Radio-nuclide ventriculography	IDC with same ECG criteria and in the absence of concomitant obstructive coronary artery disease	NR	Moderate stage	246/106	109/16	574/247	2.932	HR=3.29 (1.89–5.73)	Cox proportional hazards model multi-variate analysis
Ayub-Ferreira, 2013 [39]	Brazil	Death	1999-2010	ELISA & IFAT & HAI	Clinical trial. Chagas All stages	Similar but uninfected. Mixed aetiology, not specified	SD (14.5%), HF (22.2%)	All stages	55/287	31/29	196/1024	5.58	HR= 2.76 (1.34–5.6)	NR
Bestetti, 2013 [67]	Brazil	Death	2000-2008	Serology	Chronic systolic HF	Same criteria for systemic HTA and chronic systolic heart failure	NR	Moderate stage	244/130	185/35	1220/650	2.82	HR=2.2 (1.47–3.40)	Cox proportional hazard model adjusted for confounders
Peixoto, 2015 [22]	Brazil	Death	2005-2012	NR	Patients under cardiac resynchronization therapy; mean LVEF = 25.3	Same characteristics but Ischemic and idiopathic aetiologies	NR	Severe stage	115/311	86/111	310/839	2.10	NR (NR)	NR
Traina, 2015 [68]	USA and CA^a^	Death or HT	2007-2010	IFAT & ELISA	Cardio-myopathy with left ventricular ejection fraction (LVEF of ≤40%) and previous residence in Latin America	Any aetiology identified	NR	Moderate stage	25/110	9/11	42/209	4.07	HR=4.46 (1.8–10.8)	Un-adjusted
Sherbuk, 2015 [69]	Bolivia	Death	2012-2013	ELISA & HAI & TESA blot	From asymptomatic to severe cases	Similar stages but uninfected	NR	All stages	160/60	23/4	394/462	2.42	HR=1.78 (1.19–2.65)	NR

*ID* identification; *exp* exposed (Chagas-positive); *non-exp* non-exposed (Chagas-negative); *Crude RR* crude relative risk estimated manually form the data in the paper; *95%CI* 95% Confidence Interval; *HR* hazard ratio; *OR* odds ratio; *CF* complement fixation test; *ELISA* Enzyme-Linked Immunosorbent Assay; *HAI* hemagglutination inhibition test; *IFAT* immunofluorescent test; *TESA Trypanosoma cruzi* excreted-secreted antigens blot; *LVEF* left ventricular ejection fraction; *ECG* electrocardiogram; *RBBB* right bundle branch block; *IDC* idiopathic dilated cardiomyopathy; *HF* heart failure; *HT* heart transplant; *HTA* arterial hypertension; *SD* sudden death; *GenP* general population; *NYHA* New York Heart Association Functional Classification; *NR* not reported. ^a^ *CA* Central America 90%. The references are as appear on the main text

### Pooled estimates

**Fig. 2:**
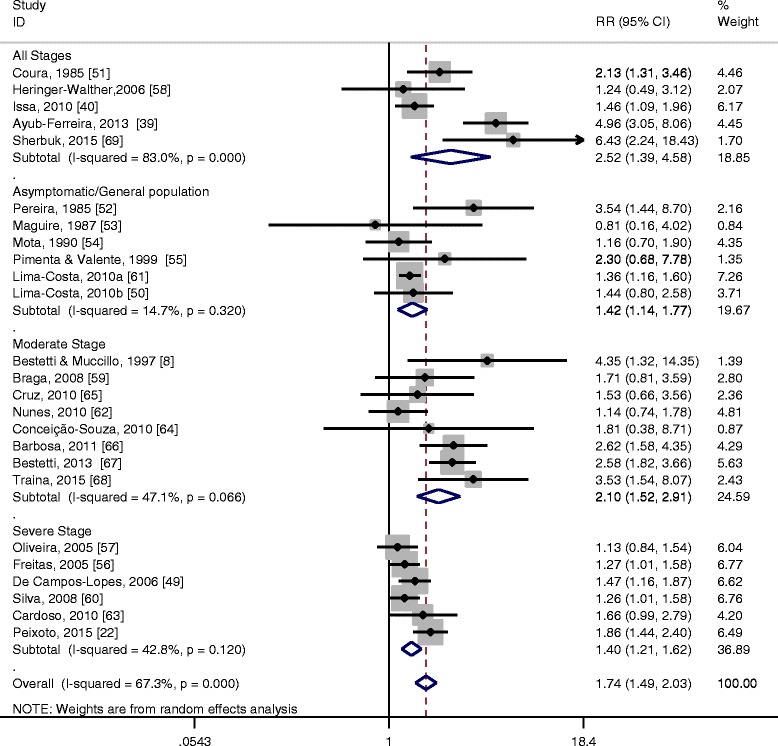
Forest plot of the meta-analysis conducted using a random-effects model to quantify excess mortality in Chagas versus non-Chagas individuals. (Reference numbers are cited as in the main text)

When pooling all studies, the overall RR was 1.74 (95 % CI 1.49–2.03. This RR reflects the overall excess mortality for chagasic patients compared with similar controls regardless of their clinical presentation. The corresponding overall annual mortality rate (AMR) was 0.18 among the chagasic groups versus 0.10 among the control groups. The observed I^2^ statistic showed substantial heterogeneity among studies (I^2^ = 67.3 %, *τ*^2^ = 0.07, *p* < 0.01) (Fig. 2). Results were similar for a fixed-effects model (Additional file 2: Figure S1) confirming the robustness of our conclusions. The value of ARP above the overall background mortality rate was estimated to be 42.5 %.

### Exploring heterogeneity

The contribution of study heterogeneity varied between clinical groups, with the greatest heterogeneity being exhibited in the clinical presentation category that included all stages (I^2^ = 83 %, *p* < 0.01). The heterogeneity present in the moderate and severe category was lower and not statistically significant (I^2^ = 47.1 %, *p* = 0.07 and I^2^ = 42.8 %, *p* = 0.12 respectively). The lowest degree of heterogeneity was present among the asymptomatic cases (14.7 %, *p* = 0.32). Figure 2 and Additional file 2: Table S3 present detailed results.

While there was clear evidence of differences in terms of AMRs between disease groups, the magnitude of excess mortality among chagasic patients, measured by the RRs, did not differ significantly between clinical groups. For the severe clinical group, AMR was 0.43 in the patients with Chagas versus 0.29 in the non-Chagas patients (RR = 1.40, 95 % CI 1.21–1.62). For the moderate clinical group the corresponding AMR values were 0.16 versus 0.08 (RR = 2.10, 95 % CI 1.52–2.91) and for the asymptomatic/general population category AMR was 0.02 (in Chagas disease patients) versus 0.01 in non-(RR = 1.42, 95 % CI 1.14–1.77).

Meta-regression on other covariates showed no evidence of significant confounding factors when adjusting for clinical classification, starting year of the study, proportion of males included, and location (country) of the study. However, sufficient information to conduct the meta-regression analysis was only available in 19 out of 25 selected studies. These results are summarised in Additional file 2: Table S4. No further investigation of the impact of sub-groups was feasible due to the small number of studies available.

### Publication bias analysis and sensitivity analysis

**Fig. 3:**
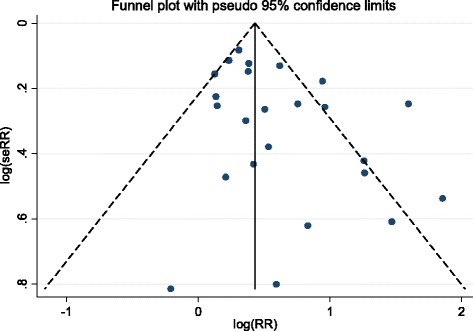
Funnel plots of the logarithm of Relative Risk (log(RR)) for Egger’s test of publication bias

Publication bias was explored firstly by looking at funnel plots (Fig. 3). The relative lack of symmetry among small-sample studies indicated a potential bias, consistent with small studies failing to report negative results. Although this bias was not significant using the Egger’s test (*p* = 0.08), we used trim-and-fill methodology (Additional file 2: Figures S2 and S3) to correct for this potential bias, re-estimating the overall excess mortality. Initial estimates were robust to publication bias, and after correction the overall RR decreased only slightly to 1.42 95 % CI 1.19–1.70.

In the sensitivity analysis, the point and uncertainty estimates of the RR remained unaffected by removing in turn a single study (Additional file 2: Figure S4), leading to the conclusion that no outliers were present in our study selection. Finally, using only the “high quality” papers yielded a RR = 2.07 (95 % CI 1.54–2.78), emphasizing the robustness of our results and suggesting a low impact of the quality of studies on the estimated excess mortality values (Additional file 2: Figure S5).

## Discussion

Our study is the first to review, collate and analyse available (published) studies on mortality rates associated with Chagas disease using a robust and coherent meta-analytical framework. One of the motivations for this meta-analysis was to assess whether Chagas disease induces a higher risk of mortality compared to control populations with similar clinical symptoms. The overall excess mortality, estimated as a relative risk ratio, was equal to 1.74 (95 % CI 1.49–2.03), which was robust to both publication bias (RR = 1.42, 95 % CI 1.19–1.70) and highest quality studies (RR = 2.07, 95 % CI 1.54–2.78).

Interestingly, this (statistically significant) magnitude of excess mortality appeared to affect equally patients with mild, moderate or severe symptoms (relative to their baseline, background mortality). By contrast, AMRs increased with clinical severity, from 0.02 in asymptomatics to 0.43 in those with severe symptoms (in Chagas patients), whereas AMR values ranged from 0.01 in asymptomatics to 0.29 in those with severe disease but without Chagas disease.

We found no significant impact of sex and study location (country) on excess mortality. However, larger studies that have focused only on cohorts of chagasic patients, consistently report men as being at increased risk of cardiac disease and death [9, 36]. Country of study may reflect a differential distribution of *T. cruzi* genotypes, and these are believed to influence disease progression and hence mortality [37]. The fact that no effect of country was found in our study may be due to the relatively limited number of studies with informative data available and their small sample sizes. It may also be possible that although disease progression rates may indeed be influenced by country (proxy for *T. cruzi* genotypes), excess mortality is not, once a given clinical/symptomatic stage has been reached.

Some researchers have suggested that the poorer prognosis in chagasic patients compared to that in patients with other aetiologies, under a similar ventricular function, may be driven by the occurrence of malignant ventricular arrhythmias and consequently sudden death [38]. However, there is strong evidence suggesting that progressive heart failure has become more important than sudden death as the most common mode of death in Chagas disease, mainly as a consequence of the introduction of better anti-arrhythmic therapy, such as beta-blockers and devices [39]. Some studies suggest that the introduction of beta-blocker agents has attenuated the poor outcome of chagasic patients, making it similar to that of patients with other cardiac aetiologies [40]. We conjectured that this gradual therapeutic improvement could have influenced temporal trends of mortality in the two groups under comparison (Chagas and non-Chagas disease patients) [39, 41]. We tested this hypothesis by exploring whether the effect size estimated changed according to the starting year of the study―a proxy for potential improvement in prognosis derived from the introduction of better treatments over time. The meta-regression analysis did not find statistical differences between the two groups regarding this covariate, but this lack of evidence may be due to the paucity of good quality studies investigating predictors of mortality in Chagas disease patients and control groups.

Our study provides a strong evidence base to help inform the understanding of Chagas-associated mortality rates by researchers and practitioners in the field of Chagas disease. Thus far, although numerous data sources exist, no consensus on the operation and/or magnitude of mortality rates due to Chagas disease has been reached. Some studies looking at the dynamics of Chagas disease assume an excess mortality for a named determinate stage (i.e. rates of 0.10) [15], whereas others simply ignore the excess mortality [42]. A recent review by Nouvellet et al. [36], exploring different modelling approaches for Chagas disease, reported a lack of agreement between current models regarding inclusion of virulence and mortality due to *T. cruzi* infection, with mortality rates attributed to Chagas disease ranging between 0 and 0.30, usually assigned to the final clinical stage of the disease [43]. Studies assessing the burden of disease and the cost effectiveness of interventions [2, 16, 44] have considered excess mortality, with values ranging from 0.04 to 0.3, depending on whether heart failure is included. These assumptions are critically relevant when linking transmission dynamics models and measures of incidence [45] to disease (morbidity-mortality) models in order to estimate burden of disease, calculate the contribution to DALYs due to Chagas, and quantify the cost-effectiveness of interventions. (see [46, 47] for an example of this crucial process in another NTD).

Our study indicates that much of the heterogeneity in the mortality rates quoted and used in the literature stems from a lack of agreement on clearly-defined disease stages. This results in mortality rates being calculated from already heterogeneous populations, leading to further confusion between rates of disease progression and mortality. By classifying Chagas patients using standard clinical stages used for other heart disease conditions (e.g. the NYHA classification), we attempted to bring coherence in the way disease progression and mortality can be defined and assessed. When evaluating Chagas disease burden, it will be necessary not only to consider the rates of Chagas-associated mortality (quantified here) but also the likely increased probability with which *T. cruzi*-infected patients progress from asymptomatic to moderate and to severe symptoms (e.g. NYHA I–II and NYHA III–IV respectively). While heart conditions typically develop with age, *T. cruzi*-infected patients are likely to develop heart disease earlier in their lives. Therefore, premature death due to Chagas must account for both the excess mortality in a particular clinical stage and the increased probability of progressing to such stage. While this study concerns the former, further research is needed to address the latter within a coherent framework to characterise rigorously rates of clinical progression in Chagas disease.

Finally, a large body of evidence suggests that socioeconomic status also influences the prognosis of cardiovascular disease [48]. A study investigating socioeconomic conditions and mortality in Brazil has shown that Chagas disease acts as a (clinical) predictor alongside socioeconomic situation [49]. However, as Chagas disease is mostly prevalent in poor populations, the independent contribution of these two factors and their interaction regarding the risk of mortality may be difficult to disentangle. One of the studies included in this review [43] found Chagas disease to be a predictor of all-cause mortality independently of socio-economic status [49]. Another study [50] found Chagas disease to be a predictor of mortality due to stroke after adjusting for educational status among other variables, with a HR = 2.25 (95 % CI 1.25–4.44), suggesting that Chagas disease is an independent contributor to mortality.

## Conclusions

The systematic review and meta-analysis conducted in this study identified a consistent body of evidence indicating that Chagas disease is associated with statistically significant excess mortality. The relative risk was 1.74 (95 % CI 1.49–2.03) and the attributable risk percent was 42.5 %. This excess mortality affected all Chagas disease patients regardless of their clinical presentation. Annual mortality rates increased with clinical severity. These results were robust to publication bias and variations in study quality. Heterogeneity in published mortality rates, and/or lack of recognition of the excess mortality, is likely to be due to heterogeneity (or absence) of clinical stage classification. Therefore, we advocate the use of a standardised system of disease severity such as the NYHA grading system (used here). Adoption of a well-characterised classification system will also help in the much needed estimation of rates of disease progression associated with *T. cruzi* infection. Our results have implications for the mathematical modelling, burden of disease estimation and economic evaluations of American trypanosomiasis and its control.

### Ethics approval and consent to participate

Not applicable.

## Additional files

Additional file 1:
**PRISMA Checklist.** (PDF 65 kb) Additional file 2:
**Details on Methods and Analyses.** (PDF 80 kb)

## Abbreviations

AMR: annual mortality rate
ARP: attributable risk percent
CI: confidence interval
DALY: disability-adjusted life year
df: degrees of freedom
ECG: electrocardiogram
HR: hazard ratio
NOS: Newcastle-Ottawa Scale
NTD: neglected tropical disease
NYHA: New York Heart Association Functional Classification
OR: odds ratio
PRISMA: Preferred Reporting Items for Systematic Reviews and Meta-Analyses
RR: relative risk (or risk ratio)
YLL: years of life lost
