# Supplementary material for: Increased mortality attributed to Chagas disease: a systematic review and meta-analysis
Source: Parasit Vectors. 2016 Jan 27;9:42. doi: 10.1186/s13071-016-1315-x (PMC4728795; doi:10.1186/s13071-016-1315-x)
Supplement: Additional file 2: — Details on Methods and Analyses. (PDF 80 kb) [file 13071_2016_1315_MOESM2_ESM.pdf]

Additional File 2. Details on Methods and Analyses

Increased mortality attributed to Chagas disease: a systematic review and meta-analysis

Zulma M. Cucunubá[1,2], Omolade Okuwoga[1], María-Gloria Basáñez[1,§], Pierre Nouvellet[1,3,§]

1. London Centre for Neglected Tropical Disease Research (LCNTDR), Department of Infectious Disease Epidemiology, School of Public Health, Faculty of Medicine (St Mary's campus), Imperial College London, London, United Kingdom
2. Grupo de Parasitología – RED CHAGAS, Instituto Nacional de Salud, Bogotá, Colombia
3. Medical Research Council Centre for Outbreak Analysis and Modelling, Department of Infectious Disease Epidemiology, School of Public Health, Faculty of Medicine (St Mary's campus), Imperial College London, London, United Kingdom

Corresponding author: Zulma M. Cucunubá (E-mail: zcucunuba@gmail.com). Department of Infectious Disease Epidemiology, School of Public Health, Faculty of Medicine (St Mary's campus), Imperial College London, Norfolk Place, London W2 1PG, United Kingdom.
Tel: + 44 0(20) 75943229

[§]Joint last authors

Authors e-mail addresses:

Zulma M. Cucunubá	zcucunuba@gmail.com; zmc13@imperial.ac.uk
Omolade Okuwoga	omolade.okuwoga14@imperial.ac.uk
María-Gloria Basáñez	m.basanez@imperial.ac.uk
Pierre Nouvellet	p.nouvellet@imperial.ac.uk

Contents

1. Search strategy.......... 3
2. Quality assessment.......... 5
3. Fixed-effects model.......... 9
4. Heterogeneity analysis.......... 10
5. Meta-regression.......... 11
6. Publication bias.......... 12
7. Sensitivity analysis.......... 14

1. Search strategy

Different algorithms were designed to search for the relevant literature; the final algorithms used for the search in the different datasets up to 30th September 2015, are presented in Table S1.

Table S1. Algorithms and databases used for the association between Chagas disease and mortality

Database	Algorithm	Number of titles	Specifications used
PubMed and MEDLINE	((((((mortality) OR death)) OR (((((progression) OR outcome) OR follow up) OR long term) OR prognos*)) OR (((((survival) OR cohort) OR clinical trial) OR hazard*) OR prospective))) AND ((((Chagas[Title/Abstract]) OR "Trypanosoma cruzi"[Title/Abstract]) OR chagas disease[MeSH Terms]) OR American trypanosomiasis)	2,242	Humans only filter
EMBASE	1. Chagas disease/ or Chagas.mp.<br>2. trypanosoma cruzi.mp. or Trypanosoma cruzi/<br>3. american trypanosomiasis.mp.<br>4. 1 or 2 or 3<br>5. mortality/ or mortality.mp.<br>6. death.mp. or death/<br>7. 5 or 6<br>8. progression.mp.<br>9. outcome.mp.<br>10. follow-up.mp.<br>11. long-term.mp.<br>12. prognos*.mp. [mp=title, abstract, heading word, drug trade name, original title, device manufacturer, drug manufacturer, device trade name, keyword]<br>13. 8 or 9 or 10 or 11 or 12<br>14. survival.mp.<br>15. cohort.mp.<br>16. clinical trial.mp. [mp=title, abstract, heading word, drug trade name, original title, device manufacturer, drug manufacturer, device trade name, keyword]<br>17. hazard*.mp. [mp=title, abstract, heading word, drug trade name, original title, device manufacturer, drug manufacturer, device trade name, keyword]<br>18. 14 or 15 or 16 or 17<br>19. 7 or 13 or 18<br>20. 4 and 19	2,837	Humans only filter

	21. prospective.mp.<br>22. 14 or 15 or 16 or 17 or 21<br>23. 7 or 13 or 22<br>24. 4 and 23		
Web of Science	((Chagas) OR (Trypanosoma cruzi) OR (American trypanosomiasis)) AND ((mortality) OR (death) OR (survival) OR (cohort) OR (clinical trial) OR (hazard) OR (prospective) OR (progression) OR (outcome) OR (follow up) OR (long term) OR (prognos*))	1,705	Humans only Search AND (humans OR patients OR cases)
LILACS	(tw:((chagas) OR (trypanosoma cruzi) OR (enfermedad de chagas) OR (doença de chagas) OR (American trypanosomiasis))) AND ((tw:(mortality OR mortalidad OR mortalidade OR death OR muerte OR morte)) OR (tw:(Progression OR Progresión OR Progressão OR Evolução da cardiopatia OR Outcome OR Desenlace* OR desfecho*)) OR (tw:(Survival OR Supervivencia OR Sobrevivência OR Follow-up OR Seguimiento OR Seguimento OR prospective OR prospective OR longitudinal OR Cohort* OR coort* OR prognos* OR pronóstico OR prognóstico OR (clinical trial) OR (ensayo clínico) OR (ensaio clínico) OR hazard)))	2,151	Humans only filter
**TOTAL**		**8,935**	

2. Quality assessment

The Newcastle–Ottawa Scale (NOS) for quality assessment of cohort studies was used (http://www.ohri.ca/programs/clinical_epidemiology/oxford.asp). Below, we describe the criteria used for this scale in the context of our cohort studies on Chagas disease.

Note: A study can be awarded a maximum of one star for each numbered item within the selection and outcome categories. A maximum of two stars can be given for comparability

Selection

1) Representativeness of the exposed cohort – **Chagas disease patients with clear definitions and tests to be classified in one of these clinical groups:**
- Asymptomatic/general population: asymptomatic or minimal ECG findings or from the general community (survey)
- Severe stage: proved by NYHA class III or IV
- Moderate Stage: proved by NYHA class III or IV
- All stages: studies with patients in several groups

a) truly representative of the in the community **(Star)**
b) somewhat representative of the average in the community **(Star)**
c) selected group of users, e.g. nurses, volunteers
d) no description of the derivation of the cohort

2) Selection of the non-exposed cohort – **Non-Chagas disease patients with clear definitions and tests to be classified in the same corresponding clinical groups:**

a) drawn from the same community as the exposed cohort **(Star)**
b) drawn from a different source
c) no description of the derivation of the non-exposed cohort

3) Ascertainment of exposure – **Chagas disease diagnosis**
a) secure record (e.g. surgical records) **(Star)**
b) structured interview **(Star)**
c) written self-report
d) no description

4) Demonstration that outcome of interest **(death)** was not present at start of study
a) yes **(Star)**
b) no

Comparability

1) Comparability of cohorts on the basis of the design or analysis
a) study controls for **gender and/or age** **(Star)**
b) study controls for any additional factor **(Star)** (This criterion could be modified to indicate specific control for a second important factor.)

Outcome

1) Assessment of outcome **– mortality: clear description of how the outcome was investigated**
a) independent blind assessment **(Star)**
b) record linkage **(Star)**

c) self-report
d) no description

2) Cohort was follow up long enough for outcomes to occur – **at least 1 year**
a) yes (select an adequate follow up period for outcome of interest) **(Star)**
b) no

3) Adequacy of follow-up cohorts
a) complete follow up - all subjects accounted for **(Star)**
b) subjects lost to follow up unlikely to introduce bias - small number lost - > 10% follow up, or description provided of those lost) **(Star)**
c) follow-up rate < 10% and no description of those lost to follow up
d) no statement

Table S2. Newcastle–Ottawa Scale for quality assessment of the studies included in the meta-analysis

First author [Reference]	Year	Study type	Follow-up type	1st Star<br><br>Represen-tativeness of the studied population	2nd Star<br><br>Cohort from same community	3rd Star<br><br>Ascertain-ment of exposure (diagnostic test)	4th Star<br><br>Outcome is not present at the beginning	5th Star<br><br>Compar-ability of cohorts Controlled for age and sex	6th Star<br><br>Controlled for other factors	7th Star<br><br>Ascertain-ment of outcome (death)	8th Star<br><br>(At least 1 year of follow up)	9th Star<br><br>l Lost to follow-up <10%	Total Stars	Overall Quality
Coura [1]	1985	Cohort	Prospective	1	1	1	1	1	0	1	1	0	7	High
Pereira [2]	1985	Cohort	Prospective	1	1	1	1	1	0	1	1	0	7	High
Maguire [3]	1987	Cohort	Prospective	1	1	1	1	1	0	1	1	1	8	High
Mota [4]	1990	Cohort/	Prospective	1	1	1	1	1	0	1	1	0	7	High
Bestetti [5]	1997	Cohort	Prospective	1	1	0	1	1	0	0	1	0	5	Moderate
Pimenta [6]	1999	Cohort	Prospective	1	1	1	1	0	0	1	1	0	6	Moderate
Freitas [7]	2005	Cohort	Retrospective	1	1	0	1	0	0	1	1	0	5	Moderate
De Oliveira [8]	2005	Cohort	Prospective	1	1	1	1	0	0	1	0	1	6	Moderate
De Campos Lopes [9]	2006	Cohort	Prospective	1	1	1	1	1	0	1	1	0	7	High
Heringer-Walther [10]	2006	Cohort	Prospective	1	1	0	1	1	1	0	1	0	6	Moderate
Braga [11]	2008	Cohort	Prospective	1	1	0	1	1	1	0	0	0	5	Moderate
Silva [12]	2008	Cohort	Prospective	1	1	0	1	0	0	0	1	1	5	Moderate
Lima-Costa A [13]	2010	Cohort	Prospective	1	1	1	1	1	0	1	1	0	7	High
Lima-Costa B [14]	2010	Cohort	Prospective	1	1	1	1	1	1	1	1	1	9	High
Pereira-Nunes [15]	2010	Cohort	Prospective	1	1	1	1	1	1	1	1	1	9	High
Issa [16]	2010	Cohort	Prospective	1	1	0	1	1	0	0	0	0	4	Low

Cardoso [17]	2010	Cohort	Prospective	1	1	0	1	1	0	0	1	1	6	Moderate
Conceição-Souza [18]	2010	Cohort	Prospective	1	1	0	1	1	1	0	1	0	6	Moderate
Cruz [19]	2010	Cohort	Prospective	1	1	1	1	1	0	0	1	0	6	Moderate
Barbosa [20]	2011	Cohort	Prospective	1	1	1	1	1	0	0	1	0	6	Moderate
Ayub-Ferreira [21]	2013	Cohort	Prospective	1	1	1	1	1	0	1	1	1	8	High
Bestetti [22]	2013	Cohort	Prospective	1	1	1	1	1	0	0	1	1	7	High
Peixoto [23]	2015	Cohort	Prospective	1	1	1	1	1	0	1	1	0	7	High
Traina [24]	2015	Cohort	Prospective	1	1	1	1	1	0	1	1	1	8	High
Sherbuk [25]	2015	Cohort	Retrospective	1	1	0	1	0	0	0	1	0	4	Low

Fixed-effects model

A fixed-effects model was run for comparison with the random-effects model presented in the main text (Fig. 2). The assumption for this model is that the effect size varies between studies due to sampling error (error in estimating the effect size). In this case, the weight of the study is mainly driven by the sample size.

Figure S1. Forest plot of the fixed-effects model for the association between Chagas disease and mortality

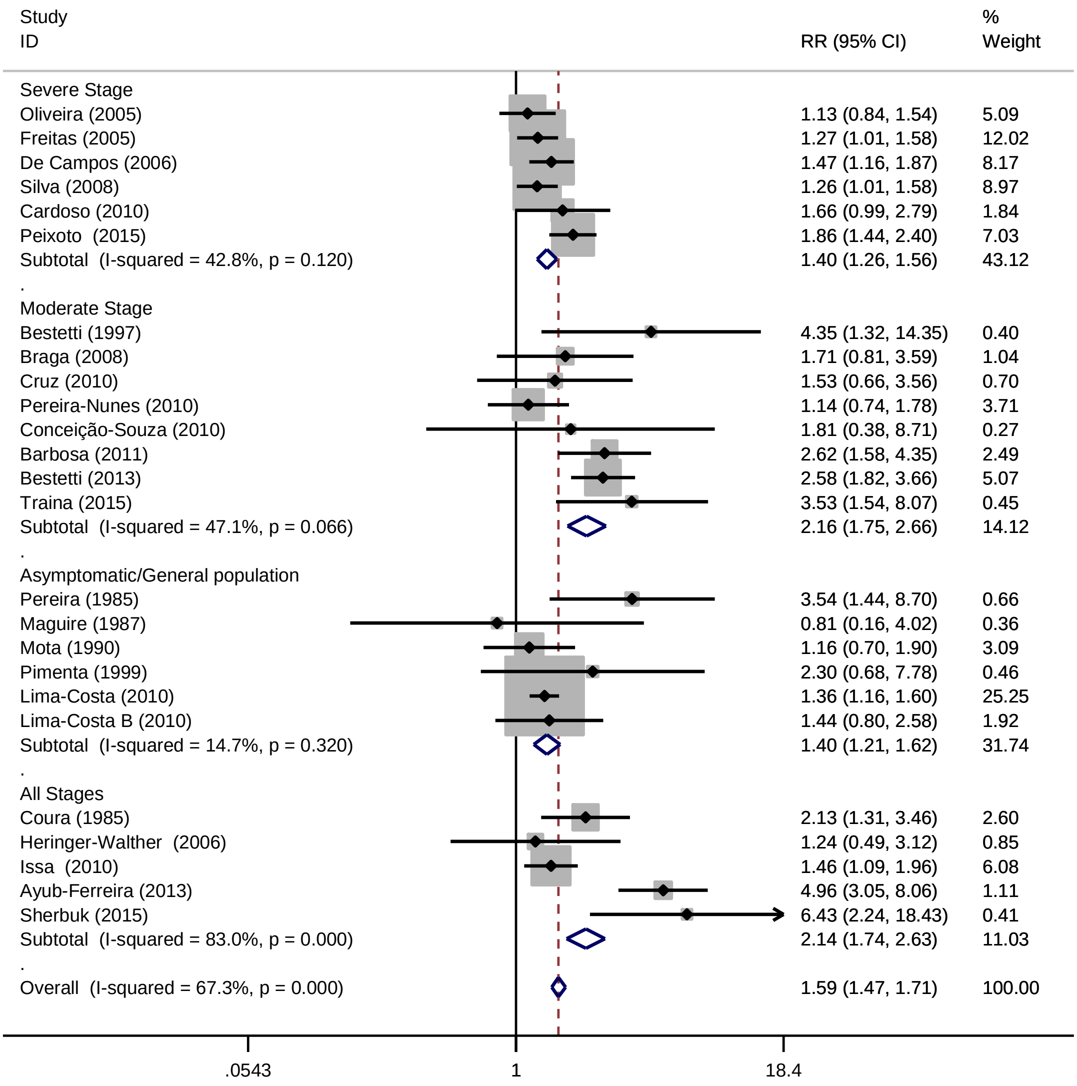

3. Heterogeneity analysis

Heterogeneity among studies was measured using Cochran's Q test and I^2 statistic [26]. Cochran's Q is calculated by adding the squared deviations of each study's estimate from the overall meta-analytic estimate, weighting each study's contribution. For the results of the random-effects model presented here and in the main text, those weights account not only for the sample size of the particular study, but also for the variance between studies (tau-squared) within the particular group (i.e. the clinical categories or random effect). The p-values for this test are obtained by comparing the Q statistic with a chi-square distribution with k-1 degrees of freedom (where k is the number of studies). The I^2 statistic describes the percentage of variation across studies that is due to study heterogeneity rather than chance (a measure of the degree of inconsistency in the studies' results), $I^2 = 100\% \times (Q–df)/Q$, where Q is the Cochran's chi-squared statistic and df its degrees of freedom [26].

The most common guide to interpreting I^2 is as follows:

- 0% to 40%: heterogeneity may not be important
- 30% to 60%: may represent moderate heterogeneity
- 50% to 90%: may represent substantial heterogeneity
- 75% to 100%: represents considerable heterogeneity

In our analysis, heterogeneity was mostly present in specific clinical groups (Table S3).

Table S3. Results of heterogeneity tests in both fixed- and random-effects models

**Clinical classification**	**Cochran's Q test**	**df**	**p-value**	**I^2 statistic**	**Fixed-effects model**		**Random-effects model**		
					**Significant test of RR=1**		**Tau-squared**	**Significant test of RR=1**	
					**z**	**p-value**		**z**	**p-value**
Severe	8.74	5	0.120	42.8%	6.14	<0.001	0.014	4.51	<0.001
Moderate	13.24	7	0.066	47.1%	7.24	<0.001	0.09	3.12	0.002
Asymptomatic/ general population	5.86	5	0.320	14.7%	4.56	<0.001	0.014	3.12	0.002
All stages	23.59	4	0.000	83.0%	7.24	<0.001		3.04	0.002
Overall	73.37	24	0.000	67.3%	12.08	<0.001	0.079	6.98	<0.001

4. Meta-regression

Meta-regression formally compares the differences in event rates across the selected study-level covariates and estimates the among-study variance, allowing the effects of multiple factors to be investigated simultaneously. In meta-regression, the outcome variable is the effect estimate, so the regression coefficient obtained will describe how the outcome variable (RR in this case) changes with a unit increase in the explanatory variable (the potential effect modifier) [27].

For this study a meta-regression was used to test the impact of potential effect modifiers, namely, clinical characteristics, follow-up time, starting year of the study, and proportion of men, on effect size of the RR (Table S4).

Table S4. Meta-regression on the RR effect of Chagas on mortality (measured as logRR).

logRR	Coefficient	Standard Error	*t*	p>|*t*|	95% Confidence Interval	
**Clinical classification**	0.32	0.18	1.76	0.10	–0.07	0.70
**Star year of the study**	0.01	0.01	0.74	0.47	–0.02	0.04
**Proportion of men**	–2.11	1.33	1.59	0.14	–4.96	0.74
**Location**	–0.22	0.27	0.83	0.42	–0.80	0.35
**Constant**	–19.47	28.89	0.67	0.51	–81.44	42.49

5. Publication bias

To explore the potential impact of publication bias on our estimates of excess mortality, we used a trim-and-fill technique to re-estimate the effect incorporating hypothetically missing studies [28]. The results are presented in Fig. S3 and Fig. S4.

Figure S2. Funnel plot of trim-and-fill technique for re-estimating the effect size

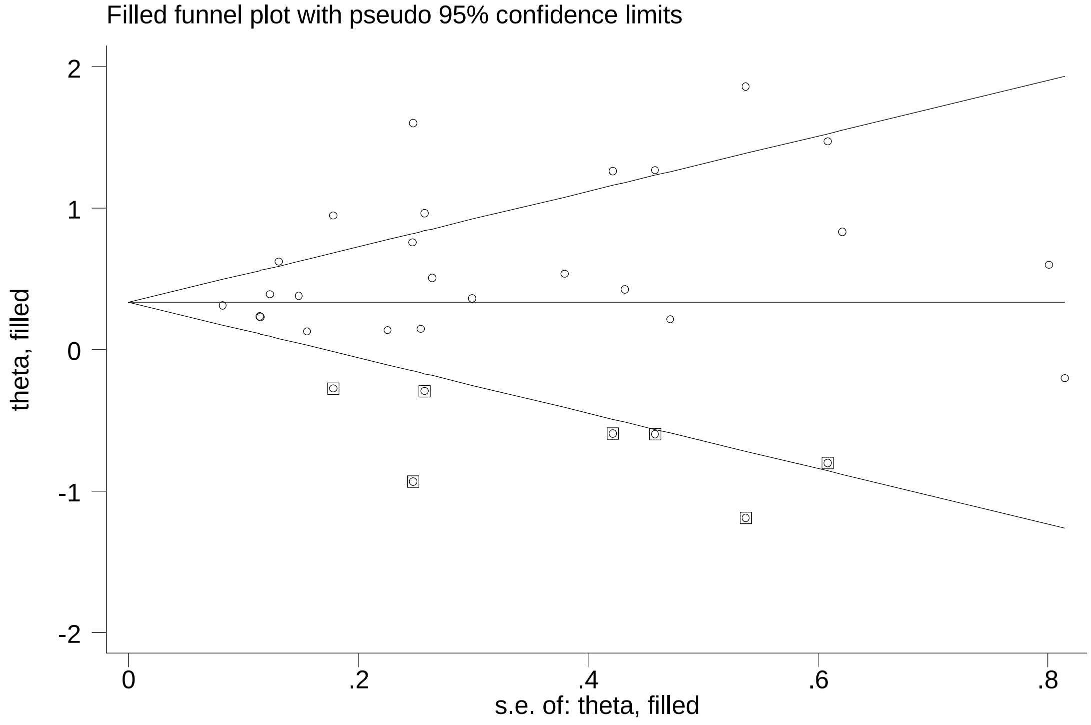

On the left axis the log(RR) (theta) from each study is plotted against its corresponding standard error (s.e.). Circles represent the studies included in the meta-analysis and the squares represent the hypothetically missing studies. Lines represent pseudo 95% confidence limits for the re-estimate using the 'filled' studies.

Figure S3. Forest plot for the trim-and-fill technique

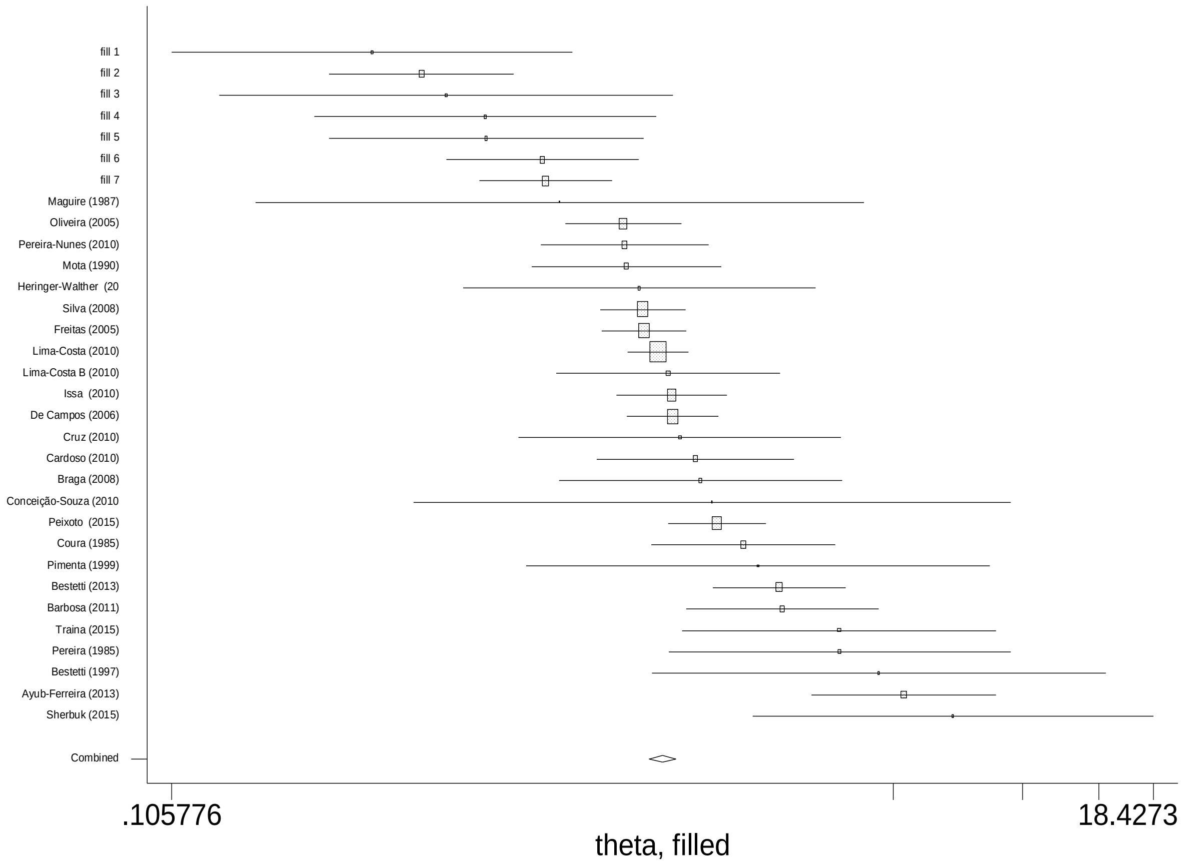

The effect estimate (RR, theta) from the studies included in the meta-analysis (with authors and years) and the hypothetically missing studies (fill 1, 2, 3, 4, 5, 6, 7) are plotted. The missing studies would have a bias towards lower excess mortality.

The magnitude of the RR re-estimated after applying the Trim and Fill technique (as a random-effects model) is 1.42 (95%CI 1.19–1.70).

This result indicates that the RR estimate and its uncertainty bounds obtained from the meta-analysis are not strongly affected by missing studies.

6. Sensitivity analysis

To identify potential outliers and assess their potential impact on the results of the meta-analysis, we re-evaluated the (fixed-effects) model after removing each study one at a time. The original RR estimate was compared to that obtained after omitting each study (Figure S4).

Figure S4. Sensitivity analysis for the fixed-effects model based on omitting one study at a time

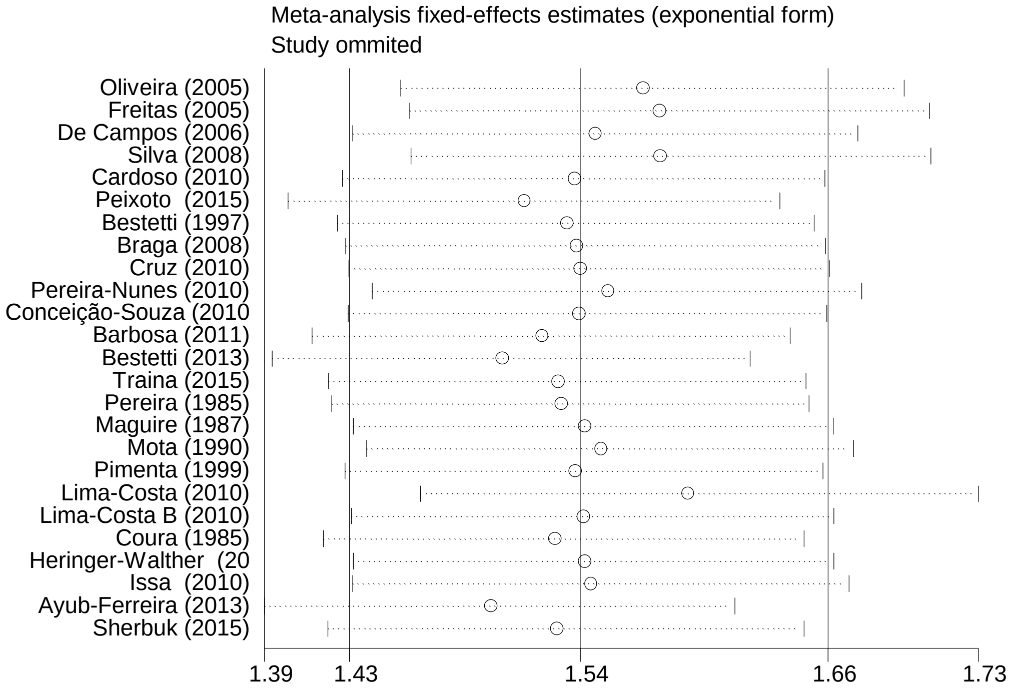

The re-estimated effect size (RR, open circles) and its 95% confidence interval (dotted horizontal lines) are plotted on the horizontal axis after removing each study in turn (left axis). Values and 95%CI were hardly affected by removing any single study, indicating no evidence of outliers in the studies selected for meta-analysis.

Figure S5 presents the results of restricting the analysis to only those ranked as 'high quality' studies (see Table S2). This sub-analysis was conducted to obtain robust estimates of excess mortality among clinical categories, and to assess the impact on our results of study quality. The results of the meta-analysis conducted using only high quality papers were very similar to those of the meta-analysis presented in the main text, indicating that study quality did not strongly influence the results.

Fig. S5. Forest plot of the fixed-effects model for the association between Chagas disease and mortality including only "high quality" studies

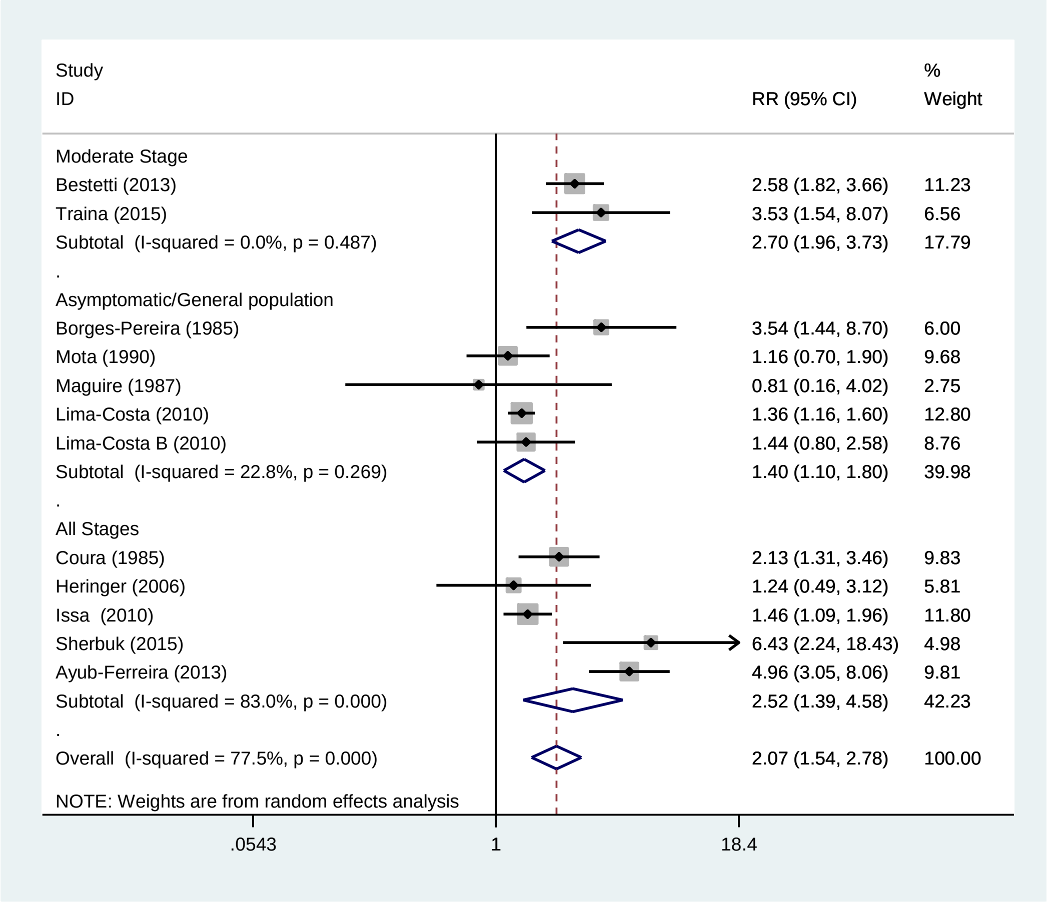

References

1. Coura JR, de Abreu LL, Pereira JB, Willcox HP. [Morbidity in Chagas' disease. IV. Longitudinal study of 10 years in Pains and Iguatama, Minas Gerais, Brazil]. Mem Inst Oswaldo Cruz. 1985; 80:73–80 (In Portuguese).

2. Pereira JB, Willcox HP, Coura JR. [Morbidity in Chagas' disease. III. Longitudinal study of 6 years, in Virgem da Lapa, MG, Brazil]. Mem Inst Oswaldo Cruz. 1985; 80:63–71 (In Portuguese).

3. Maguire JH, Hoff R, Sherlock I, Guimaraes AC, Sleigh AC, Ramos NB, et al. Cardiac morbidity and mortality due to Chagas' disease: prospective electrocardiographic study of a Brazilian community. Circulation. 1987; 75:1140–1145.

4. Mota EA, Guimarães AC, Santana OO, Sherlock I, Hoff R, Weller TH. A nine year prospective study of Chagas' disease in a defined rural population in northeast Brazil. Am J Trop Med Hyg. 1990; 42:429–440.

5. Bestetti RB, Muccillo G. Clinical course of Chagas' heart disease: a comparison with dilated cardiomyopathy. Int J Cardiol. 1997; 60:187–193.

6. Pimenta J, Valente N. Evolução clínica a longo prazo, correlacionando a presença de bloqueios da condução intraventricular em pacientes chagásicos e não chagásicos assintomáticos. Rev Soc Bras Med Trop. 1999; 32:621–631.

7. Freitas HFG, Chizzola PR, Paes ÂT, Lima ACP, Mansur AJ. Risk stratification in a Brazilian hospital-based cohort of 1220 outpatients with heart failure: role of Chagas' heart disease. Int J Cardiol. 2005; 102:239–247.

8. de Oliveira MT, Canesin MF, Munhoz RT, del Carlo CH, Scipioni A, Ramires JAF, et al. [Major clinical characteristics of patients surviving 24 months or more after hospitalization due to decompensated heart failure]. Arq Bras Cardiol. 2005; 84:161–166 (In Portuguese).

9. de Campos Lopes CB, Yamada AT, Araújo F, Pereira Barreto AC, Mansur AJ. Socioeconomic factors in the prognosis of heart failure in a Brazilian cohort. Int J Cardiol. 2006; 113:181–187.

10. Heringer-Walther S, Moreira Mda C, Wessel N, Wang Y, Ventura TM, Schultheiss H-P, et al. Does the C-type natriuretic peptide have prognostic value in Chagas disease and other dilated cardiomyopathies? J Cardiovasc Pharmacol. 2006; 48:293–298.

11. Braga JCV, Reis F, Aras R, Dantas N, Bitencourt A, Neves FS, et al. Is Chagas cardiomyopathy an independent risk factor for patients with heart failure? Int J Cardiol. 2008; 126:276–278.

12. Silva CP, del Carlo CH, de Oliveira Junior MT, Scipioni A, Strunz-Cassaro C, Ramirez JAF, et al. Why do patients with chagasic cardiomyopathy have worse outcomes than those with non-chagasic cardiomyopathy? Arq Bras Cardiol. 2008; 91:358–362.

13. Lima-Costa MF, Peixoto SV, Ribeiro ALP. Chagas disease and mortality in old age as an emerging issue: 10 year follow-up of the Bambuí population-based cohort study (Brazil). Int J Cardiol. 2010; 145:362–363.

14. Lima-Costa MF, Matos DL, Ribeiro ALP. Chagas disease predicts 10-year stroke mortality in community-dwelling elderly: The Bambui Cohort Study of Aging. Stroke. 2010; 41:2477–2482.

15. Pereira Nunes MC, Barbosa MM, Ribeiro ALP, Amorim Fenelon LM, Rocha MOC. Predictors of mortality in patients with dilated cardiomyopathy: relevance of Chagas disease as an etiological factor. Rev Esp Cardiol. 2010; 63:788–797.

16. Issa VS, Amaral AF, Cruz FD, Ferreira SMA, Guimaraes GV, Chizzola PR, et al. Beta-blocker therapy and mortality of patients with Chagas cardiomyopathy: a subanalysis of the REMADHE prospective trial. Circ Heart Fail. 2010, 3:82–88.

17. Cardoso J, Novaes M, Ochiai M, Regina K, Morgado P, Munhoz R, et al. Chagas cardiomyopathy: prognosis in clinical and hemodynamic profile C. Arq Bras Cardiol. 2010; 95:518–523.

18. Conceição-Souza GE, Pessoa-De-Melo LM, Vieira JL, Batistela V, Gorobets E, Valim LR, et al. Prognosis of Chagas’ cardiomyopathy versus non-Chagas' cardiomyopathy in beta-blockers era: 24 month follow-up. Eur Heart J. 2010; 31:130.

19. Cruz FD, Ferreira SA, Issa VS, Bocchi EA. Components of life quality evaluation in heart failure clinic. Eur J Heart Fail. 2014; 16(Suppl 2):179.

20. Barbosa AP, Cardinalli Neto A, Otaviano AP, Rocha BF, Bestetti RB. Comparison of outcome between Chagas cardiomyopathy and idiopathic dilated cardiomyopathy. Arq Bras Cardiol. 2011; 97:517–525.

21. Ayub-Ferreira SM, Mangini S, Issa VS, Cruz FD, Bacal F, Guimarães GV, et al. Mode of death on Chagas heart disease: comparison with other etiologies. A subanalysis of the REMADHE prospective trial. PLoS Negl Trop Dis. 2013; 7:e2176.

22. Bestetti RB, Otaviano AP, Fantini JP, Cardinalli-Neto A, Nakazone MA, Nogueira PR. Prognosis of patients with chronic systolic heart failure: Chagas disease versus systemic arterial hypertension. Int J Cardiol. 2013; 168:2990–2991.

23. Peixoto G, Siqueira S, Martins S, Santos J, Nishioka S, Teixeira R, et al. Cardiac resynchronization therapy in Chagas cardiomyopathy. In Europace. Volume 17; 2015:iii151.

24. Traina MI, Sanchez DR, Hernandez S, Bradfield JS, Labedi MR, Ngab TA, et al. Prevalence and impact of Chagas disease among Latin American immigrants with nonischemic cardiomyopathy in Los Angeles, California. Circ Heart Fail. 2015; 8:938–843.

25. Sherbuk JE, Okamoto EE, Marks MA, Fortuny E, Clark EH, Galdos-Cardenas G, et al. Biomarkers and mortality in severe Chagas cardiomyopathy. Glob Heart. 2015; 10:173–180.

26. Higgins JPT, Thompson SG, Deeks JJ, Altman DG. Measuring inconsistency in meta-analyses. Br Med J. 2003; 327:557–560.

27. Thompson SG, Sharp SJ. Explaining heterogeneity in meta-analysis: a comparison of methods. Stat Med. 1999; 18:2693–2708.

28. Duval S, Tweedie R. Trim and fill: a simple funnel-plot-based method of testing and adjusting for publication bias in meta-analysis. Biometrics. 2000; 56:455–463.
